# Atopic Disease and Anemia in Korean Patients: Cross-Sectional Study with Propensity Score Analysis

**DOI:** 10.3390/ijerph17061978

**Published:** 2020-03-18

**Authors:** Kiyon Rhew, Joshua D Brown, Jung Mi Oh

**Affiliations:** 1 College of Pharmacy, Seoul National University, Seoul 08826, Korea; kiyon@dongduk.ac.kr; 2 College of Pharmacy, Dongduk Women’s University, Seoul 08826, Korea; 3 Department of Pharmaceutical Outcomes and Policy, College of Pharmacy, University of Florida, Gainesville, FL 32610, USA; joshua.brown@cop.ufl.edu; 4 Research Institute of Pharmaceutical Sciences, Seoul National University, Seoul 08826, Korea

**Keywords:** allergic rhinitis, anemia, asthma, atopic dermatitis, atopic disease

## Abstract

Atopic disease is associated with chronic inflammation, and anemia has been reported in patients with inflammatory disorders such as rheumatoid arthritis, chronic obstructive pulmonary disease, and irritable bowel disease. The objective of this study was to determine whether atopic disease is associated with an increased risk of anemia. A cross-sectional study with propensity score weighting was conducted using a health insurance review agency claims dataset comprised of randomized patients who used the Korean national health system at least once in 2016. The association between atopic disease (asthma, atopic dermatitis, allergic rhinitis) and anemia (iron deficiency anemia (IDA) and/or anemia of inflammation (AI)) was examined. A total of 1,468,033 patients were included in this study. The IDA/AI prevalence was 3.1% (45,681 patients). After propensity score weighting, there were 46,958 and 45,681 patients in the non-anemic and anemic groups, respectively. The prevalence of IDA/AI in patients with atopic dermatitis, allergic rhinitis, or asthma had an odds ratio (OR) of 1.40 (95% confidence interval (CI), 1.33–1.48; *p* < 0.001), 1.17 (95% CI, 1.14–1.21; *p* < 0.001), and 1.32 (95% CI, 1.28–1.36; *p* < 0.001), respectively. In addition, the prevalence of IDA increased with higher numbers of atopic diseases. In conclusion, the prevalence of IDA/AI was higher in patients with atopic disease, even after adjusting for demographic characteristics and other risk factors. Further study is needed to distinguish between IDA and AI and to enhance understanding of the etiology of anemia in patients with inflammatory conditions.

## 1. Introduction

Anemia is one of the most common medical conditions worldwide [1,2], and a leading cause of infant morbidity and mortality. In children, anemia is associated with reduced quality of life and cognitive abnormalities [3,4]. Anemia is also associated with increased post-operative mortality in adults and overall mortality in older adults [5,6]. Atopic diseases such as atopic dermatitis, allergic rhinitis, and asthma also have a high and increasing prevalence in the general population [7,8], and they have been associated with other medical conditions, such as rheumatoid arthritis, inflammatory bowel disease, and anxiety or depression [9,10,11]. However, there has not been enough research investigating whether there is a link between anemia and atopic diseases.

Atopic diseases are associated with chronic inflammation. In patients with inflammation-related diseases, immune activation and iron deficiency can lead to anemia due to disruption of iron homeostasis [12]. Although the etiology of anemia of inflammation (AI) differs from that of iron deficiency anemia (IDA), differentiating these conditions in the typical clinical setting is difficult, as both types of anemia exhibit low hemoglobin and low iron status in blood tests [13].

Drury et al. reported an association between atopic disease and anemia in children [14]. Their study, however, did not evaluate or exclude the effects of other medical conditions correlated with both atopic disease and anemia such as rheumatoid arthritis, irritable bowel diseases, or depression. In addition, the Drury et al. study did not elucidate the mechanism underlying the association between atopic disease and anemia, but the authors suggested that IDA or anemia of chronic disease are associated with the inflammatory state characteristic of atopic diseases. Our previous study revealed that there is an association between IDA and atopic diseases in pediatric patients after adjusting patient’s confounding factors [15]. 

We hypothesized that inflammation associated with atopic disease leads to anemia, although the differential diagnosis of IDA and AI is not routinely performed. If this were the case, then there would be an association between the two diseases regardless the patient’s age. We therefore examined the association between the presence of atopic disease and IDA/AI considering the effects of medical conditions that can affect the prevalence of anemia in Korean population including all age groups. 

## 2. Materials and Methods

### 2.1. Study Subjects

The South Korean universal health coverage system, the National Health Insurance Service, covers approximately 98% of South Korean residents. As all medical information is reported to the Health Insurance Review Agency (HIRA), we used a dataset consisting of HIRA claims data for patients who used the national health system at least once in 2016 (HIRA-NPS-2016) [16]. The HIRA-NPS provides sample data for approximately 3% of the South Korean population (~1,400,000 individuals) stratified by age and gender and including data regarding demographic characteristics (age, gender, insurance policies), 1-year medical history (diagnoses, treatments, procedures, surgical history, and prescription drugs), and medical expenses. We included all patients in the dataset and excluded those with anemia other than IDA/AI and patients with veterans insurance. The Korean Standard Classification of Disease and Cause of Death-7 (KCD-7) was used for disease diagnosis.

### 2.2. Definition of Disease

If a given diagnostic code was entered in a target patients’ record once or more during the year, the patient was defined as having the corresponding disease, as diagnosed based on the KCD-7. The KCD-7 reflects updates to the International Classification of Diseases—10th revision, as recommended by the World Health Organization, concerning the subdivision classification of Korean subtype disease and the classification of Korean medical areas. The KCD-7 also provides diagnostic information for rare diseases to improve categorization and disease terminology. There are no missing values for diagnostic codes, as prescribers must enter diagnostic codes for prescribing medications or procedures in Korea, and there is no medication refill or telemedicine system. The following KCD-7 codes were used for the diagnosis of atopic diseases: Atopic dermatitis (L20), asthma (J45), and allergic rhinitis (J30.1, J30.2, and J30.3). IDA and AI were considered an anemia group; IDA was classified as code D50, and AI was classified as code D63.8. 

### 2.3. Confounders 

We included sex, age, and insurance type as socioeconomic status. Possible confounders included systemic infection (meningitis (A87, A39), bone and joint infection (BJI; M00, M01, M02, M03), sepsis (A40, A41), hepatitis (B15, B16, B17, B18, B19)), chronic kidney disease (CKD; N18), heart failure (HF; I50), diabetes mellitus (DM; E10, E11, E12, E13, E14), mental disorder (depression (F32, F33) and anxiety (F40, F41, F93, F06.4)), chronic inflammation (peptic ulcer disease (PUD; K25, K26, K27), chronic obstructive pulmonary disease (COPD; J42, J43, J44), systemic lupus erythematosus (SLE; M32), rheumatoid arthritis (RA; M05, M06, M08.0), irritable bowel disease (IBD; K51, K50)), and cancer (CXX). Confounders were chosen based on previous studies of increased risk of IDA/AI. Age (children, adolescent, adult, or elderly) and insurance type (medical health insurance or medical aid) were assessed as categorical variables. Others such as sex, insurance type, and possible confounding diseases were evaluated as dichotomous variables. 

### 2.4. Statistical Analysis

Binary logistic regression was carried out by applying inverse probability of treatment weighting (IPTW) using the propensity score. To calculate the predicting propensity score, we used a multivariable logistic regression model to estimate the probability of each patient with or without IDA/AI, including confounders (sex, age, insurance type, meningitis, BJI, sepsis, hepatitis, CKD, HF, DM, depression, anxiety, PUD, COPD, SLE, RA, IBD, and cancer). Results are presented as odds ratio (OR) with 95% confidence interval (CI). All statistical analyses were carried out using SAS 9.4 (SAS Institute Inc., Cary, NC, USA), and differences were considered statistically significant if the *P*-value was < 0.05. The study was approved by the Institutional Review Board of Dongduk Women’s University (DDWU1905-07).

## 3. Results

### 3.1. Subject Characteristics

**Figure 1:**
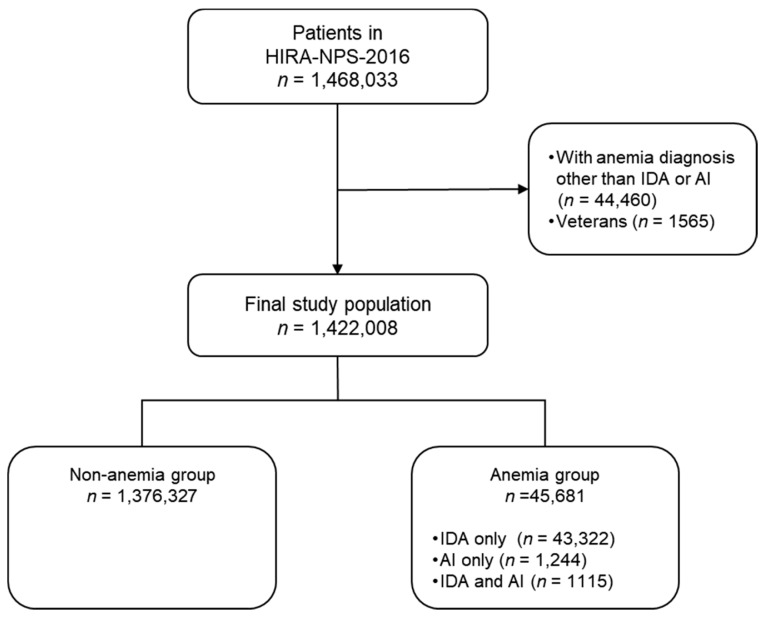
Flow diagram from study subject inclusion. HIRA: Health Insurance Review Agency, IDA: Iron deficiency anemia, AL: Anemia of inflammation.

The HIRA-NPS-2016 dataset included 1,468,033 patients. A total of 44,460 patients with conditions other than IDA/AI and 1565 patients covered by the veterans’ insurance program were excluded. Therefore, 1,422,008 patients were included in the study. Of these patients, 1,376,327 (96.8%) did not have any type of anemia, resulting in the inclusion of 46,025 patients in the anemia group. Among patients in the anemia group, 43,322 had a diagnosis of only IDA, whereas 1244 patients had a diagnosis of only AI, and 1115 were diagnosed with both IDA and AI (Figure 1). 

The baseline characteristics of the two groups are summarized in Table 1. All confounding variables differed significantly between the anemia and non-anemia groups. After application of IPTW, the baseline characteristics of the two groups were balanced (Table 1).

### 3.2. Association between Atopic Disease and IDA

The association of IDA and AI exhibited an OR of 1.40 (95% CI, 1.33–1.48; *p* < 0.001), 1.17 (95% CI, 1.14–1.21; *p* < 0.001), and 1.32 (95% CI, 1.28–1.36; *p* < 0.001) in patients with atopic dermatitis, allergic rhinitis, or asthma, respectively. Analysis of covariates indicated associations between all of the atopic diseases examined and IDA. The overall association with one atopic disease was 1.16 (95% CI, 1.13–1.20; *p* < 0.001), whereas the association was higher in those with two atopic diseases (1.40 (95% CI, 1.33–1.46; *p* < 0.001)) and increased to a > 2-fold association (OR = 2.19 (95% CI, 1.94–2.46; *p* < 0.001)) in patients with three atopic diseases. These results indicate that the overall prevalence of IDA/AI increases as the number of atopic diseases increases (Table 2). 

Compared with patients with non-atopic disease, children, adolescents, adults, males, females, and patients covered by health insurance programs who also had atopic disease exhibited an increased OR for IDA/AI. The OR for IDA/AI with allergic rhinitis was lower than that for asthma or atopic dermatitis in all of the above-mentioned patient groups. The IDA/AI OR also increased with increasing number of atopic diseases, except in adolescents. The association between atopic disease and anemia was more pronounced in pediatric patients than in any of the other age groups. In addition, the OR for anemia with atopic diseases was higher in male than female patients (Table 3).

In contrast, no associations were observed between anemia and atopic diseases or increased number of atopic diseases in elderly patients. There were no significant differences in the prevalence of anemia with any atopic disease in elderly patients compared with patients without atopic disease. Except for atopic dermatitis, there was no association between anemia and any other atopic disease in patients receiving medical aid. In addition, there was no increased prevalence of anemia with increasing number of atopic diseases in these patients.

## 4. Discussion

Atopic diseases involve inflammation, which can in turn cause AI. Our results demonstrated an association between anemia and atopic diseases such as atopic dermatitis, allergic rhinitis, and asthma. Even after the application of an IPTW approach to balance the anemia and non-anemia groups, the association between atopic diseases and IDA/AI remained. In addition, the increased prevalence of anemia with increasing number of atopic diseases supports our hypothesis that the inflammatory state of atopic diseases is associated with increased risk of anemia. 

As demonstrated in previous studies, the prevalence of anemia is higher in low-income populations [17,18]. Anemia also exhibits higher prevalence in females and elderly patients [19,20]. Our results are in agreement with those of previous epidemiology studies without weighting propensity scoring. In addition, the increased prevalence of anemia in patients with systemic infections, CKD, HF, DM, mental illness, chronic inflammatory diseases, systemic diseases, and cancer was the same as previously reported [21,22,23].

Drury et al. investigated the association between atopic diseases and anemia using a survey of pediatric and adolescent patient caregivers [14]. The present study could better support the results, as it used diagnostic codes for each disease. The Drury et al. study found no association between hay fever and anemia; however, the results of the present study showed an association between allergic rhinitis (including hay fever) and IDA. Interestingly, the OR for IDA/AI in patients with allergic rhinitis in our study was lower than that in patients with atopic dermatitis or asthma. This was the same in pediatric patients in our previous study [15]. Even though an association with atopic diseases (allergic rhinitis, atopic dermatitis, and asthma), designated the atopic match (allergic match), was shown [24,25,26], there could be differences in the magnitude of inflammation among the atopic diseases. Two previous studies reported that the more atopic diseases patients have, the higher their risk of anemia [14,15]. Our results were in agreement. This could be possible evidence of the effect of the inflammatory condition of atopic diseases on the increasing prevalence of anemia.

We found the strongest association between anemia and atopic disease in children, further supporting our hypothesis. Inflammatory diseases are chronic and commonly appear in older individuals rather than in children. Therefore, it can be assumed that the inflammatory state of atopic disease in pediatric patients more clearly demonstrates the increasing prevalence of AI. The OR for anemia in patients with atopic disease can be seen as a result of the small increase in OR with higher age. Notably, the OR for IDA/AI in adolescents with all three atopic diseases was 2.43, which was higher than in those with one or two atopic disease(s), but the 95% CI included 1. Adolescents use fewer medical services than other age groups. The number of adolescent patients diagnosed with all three atopic diseases was very small in our study, which could lead to statistical insignificance as the confidence interval widened. In addition, the prevalence of anemia in the elderly with any atopic disease did not increase. A previous study showed that proinflammatory makers including interleukin-6 and C-reactive protein were elevated in elderly populations, which could suggest a chronic and mild proinflammatory status is showed in elderly patients [27]. In other words, the proinflammatory status is already showed in elderly, so the effect of increasing the prevalence of IDA/AI in the inflammatory state of atopic diseases may be limited.

The association between atopic disease and IDA/AI was lower in female patients than male patients. Pregnancy, heavy menstrual bleeding, and blood loss during childbirth are the most common causes of IDA in women of childbearing age [28,29]. The IDA/AI group that we included may have had more isolated IDA patients in the female group than in the male group. This could be interpreted as supporting our hypothesis that atopic disease can cause AI. 

The cost of health care services in Korea is very low compared with that in other developed countries, and more than 90% of the population uses medical services at least once each year [16]. In addition, patients receiving medical aid can get most medical services at almost no cost. Several Korean studies have reported that the use of medical services and hospitalization patterns in patients receiving medical aid differ from those of patients in health insurance programs [30,31,32]. Patients receiving medical aid are hospitalized longer or use more medical services, and inappropriate medical service use and length of hospitalization were also analyzed. Our results showed that 55% of patients receiving medical aid did not receive a diagnosis of atopic disease, which means they had a diagnosis of atopic disease more frequently than other patient groups. It could be interpreted that the lack of association between atopic disease and anemia in medical aid patients is due to patterns of health service use rather than to patient characteristics.

Determination of hepcidin (HAMP) levels is important for differentially diagnosing between IDA and AI, as well as in the assessment of therapeutic options. HAMP would be low in IDA, normal in IDA/AI, and high in AI patients [33,34]. Clinicians, however, typically do not check HAMP levels to differentiate IDA and AI; IDA is usually diagnosed in atopic disease patients by a blood test. The treatment strategies for IDA and AI are also different. Oral or parenteral iron is the primary treatment for isolated IDA, but clinicians should initially focus on controlling or treating the underlying diseases in AI patients [35]. Clinicians can also expect a poor treatment response to erythropoietin-stimulating agents and oral iron in patients with high HAMP levels [36,37]. Therefore, clinicians should consider controlling inflammation to prevent anemia in atopic disease patients. It is also important to screen for anemia in patients with atopic disease in the primary care setting.

Apart from our hypothesis, several studies have suggested an association between food allergy and anemia. Several studies have shown an increased risk of eosinophilia in patients with food allergies [38,39]. There was also a correlation between atopic diseases and eosinophilic status [40]. In our study, it was very limited to determine food allergies with diagnostic code, so it could not be included in the analysis. However, it can be explained that the inflammatory status of food allergy increases the prevalence of anemia based on the study that patients with food allergies have a high risk of atopic disease and food allergy is also one of atopic diseases [41]. In addition, some studies have suggested a correlation between the maternal iron levels during pregnancy and her baby’s atopic disease including asthma or lung function [42,43,44]. Meanwhile, a recent study had shown that hemoglobin levels during the mother’s pregnancy were associated with respiratory and allergic diseases in childhood [45]. Another study suggested the increased risk of IDA in infants with cow’s milk allergy [46]. It was difficult to present a causal relationship because our study was conducted by a cross-sectional study. However, several studies presented above confirmed the correlation between atopic disease (atopic dermatitis, asthma, or food allergy) and anemia and/or iron deficiency. This may support the evidence as a result of studies that allergies cause iron deficiency at the molecular levels [47]. The causality of allergic disease and anemia and/or low iron levels cannot be accurately presented at this time, but we would like to suggest that it has an explicit association. Currently, the causality of allergic diseases and anemia and/or iron levels cannot be accurately presented, but it is suggested that there is a clear link.

This study has some limitations. First, the assumption of increased risk of AI in patients with atopic disease was indirectly shown by the association between atopic diseases and IDA/AI. There was no direct evidence that could elucidate the mechanism. In addition, as patients diagnosed with IDA were included in the analysis, the effects of patients with isolated IDA cannot be ruled out. Third, this study was designed as a cross-sectional study, so we could not determine a causal relationship between atopic disease and anemia. Nevertheless, this study has several strengths. As far as we know, this is the first study to show that atopic disease can be associated with IDA/AI in all age groups. This suggests that atopic disease could be linked with anemia, similar to other inflammatory disorders. In addition, the use of a large dataset could facilitate extrapolation to the entire Korean patient population. We also used diagnostic code for disease included not self-report. These enabled us to evaluate the association between atopic diseases and anemia more objectively.

## 5. Conclusions 

The prevalence of IDA/AI is higher in patients with atopic disease, even after adjustment for demographic characteristics and other risk factors. We also found that the more atopic diseases patients have, the higher the prevalence of anemia. This would support the hypothesis that inflammatory stats of atopic diseases, including atopic dermatitis, allergic rhinitis, and asthma increase the risk of anemia. Further study is needed to elucidate the distinction between IDA and AI in order to better understand the etiology of anemia in patients with inflammatory conditions. 

## Figures and Tables

**Table 1:** Baseline characteristics of subjects before and after inverse probability of treatment weighting.

	Before Weighting, *n* (%)		After Weighting, *n* (%)	
	No Anemia (*n* = 1,376,327)	IDA/AI (*n* = 45,681)	No Anemia (*n* = 46,958)	IDA/AI (*n* = 45,681)
Age group				
Children (<12)	160,240 (11.64)	4755 (10.41)	4769 (10.16)	4755 (10.41)
Adolescents (≥12, <18)	91,508 (6.65)	1332 (2.92)	1325 (2.82)	1332 (2.92)
Adults (≥18, <65)	948,857 (68.94)	27,093 (59.31)	27,340 (58.22)	27,093 (59.31)
Elderly (≥65)	175,722 (12.77)	12,501 (27.37)	13,524 (28.80)	12,501 (27.37)
Sex				
Male	683,856 (49.69)	14,568 (31.89)	14,705 (31.31)	14,568 (31.89)
Female	692,471 (50.31)	31,113 (68.11)	32,253 (68.69)	31,113 (68.11)
Insurance Type				
Health insurance	1,338,718 (97.27)	42,249 (92.49)	43,105 (91.80)	42,249 (92.49)
Medical aid	37,609 (2.73)	3432 (7.51)	3853 (8.20)	3432 (7.51)
Systemic infection				
Meningitis	624 (0.05)	76 (0.17)	90 (0.19)	76 (0.17)
BJI	3727 (0.27)	331 (0.72)	408 (0.87)	331 (0.72)
Sepsis	2980 (0.22)	777 (1.70)	1071 (2.28)	777 (1.70)
HEP	23,828 (1.73)	3403 (7.45)	3950 (8.41)	3403 (7.45)
CKD	4767 (0.35)	3284 (7.19)	4116 (8.77)	3284 (7.19)
HF	18,356 (1.33)	2842 (6.22)	3296 (7.02)	2842 (6.22)
DM	137,858 (10.02)	15,806 (34.60)	17,120 (36.46)	15,806 (34.60)
Mental disorder				
Depression	57,754 (4.20)	5020 (10.99)	5630 (11.99)	5020 (10.99)
Anxiety	90,513 (6.58)	7493 (16.40)	8258 (17.59)	7493 (16.40)
Chronic inflammation				
PUD	123,328 (8.96)	8839 (19.35)	9,629 (20.51)	8839 (19.35)
COPD	45,100 (3.28)	3586 (7.85)	3991 (8.50)	3586 (7.85)
SLE	1633 (0.12)	299 (0.65)	371 (0.79)	299 (0.65)
RA	24,614 (1.79)	2841 (6.22)	3286 (7.00)	2841 (6.22)
IBD	2025 (0.15)	248 (0.54)	322 (0.69)	248 (0.54)
Cancer	46,695 (3.39)	5043 (11.04)	5792 (12.33)	5043 (11.04)

IDA: Iron deficiency anemia, BJI: Bone and joint infection, HEP: Hepatitis, CKD: Chronic kidney disease, PUD: Peptic ulcer disease, COPD: Chronic obstructive pulmonary disease, SLE: Systemic lupus erythematosus, RA: Rheumatoid arthritis, IBD: Irritable bowel disease.

**Table 2:** Association between atopic diseases and anemia.

Atopic Diseases		Frequency (%)		Odds Ratio (95% CI)	*p* Value
		No Anemia (n = 46,958)	IDA/AI (n = 45,681)		
Atopic dermatitis	No	44,486 (94.73)	42,382 (92.78)	1 [Reference]	
	Yes	2472 (5.27)	3299 (7.22)	1.40 (1.33–1.48)	< 0.001
Allergic rhinitis	No	34,777 (74.06)	32,372 (70.87)	1 [Reference]	
	Yes	12,181 (25.94)	13,309 (29.13)	1.17 (1.14–1.21)	< 0.001
Asthma	No	38,907 (82.85)	35,891 (78.57)	1 [Reference]	
	Yes	8051 (17.15)	9790 (21.43)	1.32 (1.28–1.36)	< 0.001
Atopic diseases, n	0	29,193 (62.17)	26,022 (56.96)	1 [Reference]	
	1	13,247 (28.21)	13,744 (30.09)	1.16 (1.13–1.20)	< 0.001
	2	4095 (8.72)	5091 (11.14)	1.40 (1.33–1.46)	< 0.001
	3	422 (0.90)	824 (1.80)	2.19 (1.94–2.46)	< 0.001

CI: Confidence interval, IDA: Iron deficiency anemia, AI: Anemia of inflammation.

**Table 3:** Subgroup analysis of associations between atopic diseases and anemia.

Patient Characteristics	Atopic Diseases		Frequency (%)		Odds Ratio (95% CI)	*p* value
			No Anemia	IDA/AI		
Children (<12 years)	Atopic dermatitis	No	3979 (83.42)	3379 (71.06)	1 [Reference]	
		Yes	790 (16.58)	1376 (28.94)	2.05 (1.86–2.26)	<0.001
	Allergic rhinitis	No	2882 (60.44)	2319 (48.77)	1 [Reference]	
		Yes	1887 (39.56)	2436 (51.23)	1.61 (1.48–1.74)	<0.001
	Asthma	No	3010 (63.11)	1737 (36.53)	1 [Reference]	
		Yes	1759 (36.89)	3018 (63.47)	2.97 (2.74–3.23)	<0.001
	Atopic diseases, n	0	1806 (37.87)	805 (16.93)	1 [Reference]	
		1	1712 (35.90)	1662 (34.95)	2.18 (1.96–2.42)	<0.001
		2	1029 (21.58)	1696 (35.67)	3.70 (3.30-4.14)	<0.001
		3	222 (4.66)	592 (12.45)	5.98 (5.02-7.13)	<0.001
Adolescents (≥12, <18 years)	Atopic dermatitis	No	1245 (93.95)	1212 (90.99)	1 [Reference]	
		Yes	80 (6.05)	120 (9.01)	1.54 (1.15–2.06)	0.004
	Allergic rhinitis	No	951 (71.78)	869 (65.24)	1 [Reference]	
		Yes	374 (28.22)	463 (34.76)	1.36 (1.15–1.60)	<0.001
	Asthma	No	1189 (89.74)	1129 (84.76)	1 [Reference]	
		Yes	136 (10.26)	203 (15.24)	1.57 (1.25–1.98)	<0.001
	Atopic diseases, n	0	832 (62.80)	711 (53.38)	1 [Reference]	
		1	402 (30.37)	470 (35.29)	1.37 (1.16–1.62)	<0.001
		2	84 (6.32)	137 (10.29)	1.92 (1.43–2.56)	<0.001
		3	7 (0.51)	14 (1.05)	2.43 (0.97–6.13)	0.059
Adults (≥18, <65 years)	Atopic dermatitis	No	26,341 (96.35)	25,910 (95.63)	1 [Reference]	
		Yes	999 (3.65)	1183 (4.37)	1.20 (1.11–1.31)	<0.001
	Allergic rhinitis	No	20,673 (75.62)	19,710 (72.75)	1 [Reference]	
		Yes	6667 (24.38)	7383 (27.25)	1.16 (1.12–1.21)	<0.001
	Asthma	No	24,244 (88.67)	23,368 (86.25)	1 [Reference]	
		Yes	3096 (11.33)	3725 (13.75)	1.25 (1.19–1.31)	<0.001
	Atopic diseases, n	0	18,455 (67.50)	17,124 (63.20)	1 [Reference]	
		1	7108 (26.00)	7782 (28.72)	1.18 (1.136–1.23)	<0.001
		2	1677 (6.13)	2052 (7.57)	1.32 (1.233–1.41)	<0.001
		3	100 (0.37)	135 (0.50)	1.45 (1.122–1.88)	0.005
Elderly (≥65 years)	Atopic dermatitis	No	12,921 (95.54)	11,881 (95.04)	1 [Reference]	
		Yes	603 (4.46)	620 (4.96)	1.12 (1.00–1.25)	0.057
	Allergic rhinitis	No	10,270 (75.94)	9474 (75.79)	1 [Reference]	
		Yes	3254 (24.06)	3027 (24.21)	1.01 (0.95–1.07)	0.771
	Asthma	No	10,464 (77.38)	9657 (77.25)	1 [Reference]	
		Yes	3060 (22.62	2844 (22.75)	1.01 (0.95–1.07)	0.808
	Atopic diseases, n	0	8100 (59.89)	7382 (59.05)	1 [Reference]	
		1	4025 (29.76)	3830 (30.64)	1.04 (0.99–1.10)	0.119
		2	1306 (9.66)	1206 (9.65)	1.01 (0.93–1.10)	0.758
		3	93 (0.69)	83 (0.66)	0.98 (0.72–1.31)	0.867
Male	Atopic dermatitis	No	13,967 (94.98)	13,265 (91.06)	1 [Reference]	
		Yes	738 (5.02)	1303 (8.94)	1.89 (1.69–2.04)	<0.001
	Allergic rhinitis	No	11,259 (76.57)	10,411 (71.46)	1 [Reference]	
		Yes	3446 (23.43)	4157 (28.54)	1.31 (1.24–1.38)	<0.001
	Asthma	No	12,391 (84.26)	10,904 (74.85)	1 [Reference]	
		Yes	2314 (15.74)	3664 (25.15)	1.80 (1.70–1.91)	<0.001
	Atopic diseases, n	0	9618 (65.41)	8025 (55.09)	1 [Reference]	
		1	3808 (25.89)	4345 (29.83)	1.39 (1.30–1.44)	<0.001
		2	1146 (7.79)	1815 (12.46)	1.90 (1.75–2.06)	<0.001
		3	133 (0.90)	383 (2.63)	3.46 (2.83–4.22)	<0.001
Female	Atopic dermatitis	No	30,519 (94.62)	29,117 (93.58)	1 [Reference]	
		Yes	1734 (5.38)	1996 (6.42)	1.21 (1.13–1.29)	<0.001
	Allergic rhinitis	No	23,518 (72.92)	21,961 (70.58)	1 [Reference]	
		Yes	8735 (27.08)	9152 (29.42)	1.12 (1.08–1.16)	<0.001
	Asthma	No	26,516 (82.21)	24,987 (80.31)	1 [Reference]	
		Yes	5737 (17.79)	6126 (19.69)	1.13 (1.09–1.18)	<0.001
	Atopic diseases, n	0	19,575 (60.69)	17,997 (57.84)	1 [Reference]	
		1	9440 (29.27)	9399 (30.21)	1.08 (1.05–1.12)	<0.001
		2	2949 (9.14)	3276 (10.53)	1.21 (1.15–1.28)	<0.001
		3	289 (0.90)	441 (1.42)	1.66 (1.43–1.92)	<0.001
Health insurance	Atopic dermatitis	No	40,857 (94.78)	39,190 (92.76)	1 [Reference]	
		Yes	2248 (5.22)	3059 (7.24)	1.42 (1.34–1.50)	<0.001
	Allergic rhinitis	No	31,960 (74.14)	29,901 (70.77)	1 [Reference]	
		Yes	11,145 (25.86)	12,348 (29.23)	1.18 (1.15–1.22)	<0.001
	Asthma	No	36,055 (83.64)	33,293 (78.80)	1 [Reference]	
		Yes	7050 (16.36)	8956 (21.20)	1.37 (1.33–1.42)	<0.001
	Atopic diseases, n	0	27,053 (62.76)	24,130 (57.11)	1 [Reference]	
		1	12,048 (27.95)	12,657 (29.96)	1.18 (1.14–1.21)	<0.001
		2	3617 (8.39)	4680 (11.08)	1.45 (1.38–1.52)	<0.001
		3	387 (0.90)	782 (1.85)	2.27 (2.00–2.56)	<0.001
Medical aid	Atopic dermatitis	No	3629 (94.19)	3192 (93.01)	1 [Reference]	
		Yes	224 (5.81)	240 (6.99)	1.22 (1.01–1.47)	0.039
	Allergic rhinitis	No	2818 (73.13)	2471 (72.00)	1 [Reference]	
		Yes	1035 (26.87)	961 (28.00)	1.06 (0.96–1.17)	0.280
	Asthma	No	2852 (74.01)	2598 (75.70)	1 [Reference]	
		Yes	1001 (25.99)	834 (24.30)	0.91 (0.82–1.02)	0.099
	Atopic diseases, n	0	2141 (55.56)	1892 (55.13)	1 [Reference]	
		1	1199 (31.13)	1087 (31.67)	1.02 (0.93–1.14)	0.632
		2	478 (12.40)	411 (11.98)	0.97 (0.84–1.13)	0.718
		3	35 (0.92)	42 (1.22)	1.35 (0.86–2.12)	0.195

CI: Confidence interval, IDA: Iron deficiency anemia, AI: Anemia of inflammation.

## Abbreviations

***AI***	Anemia of inflammation
***BJI***	Bone and joint infection
***CI***	Confidence intervals
***CKD***	Chronic kidney disease
***COPD***	Chronic obstructive pulmonary disease
***DM***	Diabetes mellitus
***GI***	Gastrointestinal
***HIRA***	Health Insurance Review and Assessment Service
***HF***	Heart failure
***IBD***	Irritable bowel disease
***ICD-10***	International Classification of Diseases 10th Revision
***IDA***	Iron deficiency anemia
***IPTW***	Inverse probability of treatment weighting
***KCD-7***	The Korean Standard Classification of Disease and Cause of Death-7
***OR***	Odds ratio
***PUD***	Peptic ulcer disease
***RA***	Rheumatoid arthritis
***SLE***	Systemic lupus erythematosus
